# Nociceptive Threshold of Calves and Goat Kids Undergoing Injection of Clove Oil or Isoeugenol for Disbudding

**DOI:** 10.3390/ani10071228

**Published:** 2020-07-20

**Authors:** Sandra Frahm, Pierpaolo Di Giminiani, Anna Stanitznig, Julia Schoiswohl, Reinhild Krametter-Frötscher, Thomas Wittek, Susanne Waiblinger

**Affiliations:** 1 Institute of Animal Welfare Science, Department for Farm Animals and Veterinary Public Health, University of Veterinary Medicine, Vienna (Vetmeduni Vienna), Veterinärplatz 1, 1210 Vienna, Austria; sandrafrahm@gmx.de; 2 School of Natural and Environmental Sciences, Newcastle University, Newcastle upon Tyne NE1 7RU, UK; pierpaolo.digiminiani@gmail.com; 3 Department for Farm Animals and Veterinary Public Health, University Clinic for Ruminants, Vetmeduni Vienna, Veterinärplatz 1, 1210 Vienna, Austria; Anna.Stanitznig@vetmeduni.ac.at (A.S.); Julia.Schoiswohl@vetmeduni.ac.at (J.S.); Reinhild.Krametter@vetmeduni.ac.at (R.K.-F.); Thomas.Wittek@vetmeduni.ac.at (T.W.)

**Keywords:** cattle, goats, nociception, pain, disbudding, welfare

## Abstract

**Simple Summary:**

Hot-iron disbudding in calves and goat kids is a widespread husbandry procedure involving pain and, especially in goats, the risk of brain damage. The injection of clove oil under the horn bud is a potential alternative disbudding method. Clove oil, with its active substance eugenol, is cytotoxic but has anaesthetic effects, and the injection of clove oil, or the pure substance isoeugenol, under the horn bud to stop horn growth may have potential welfare benefits compared to hot-iron disbudding. We compared the injection of clove oil with the injection of isoeugenol under the horn bud with respect to pain sensitivity in this area in the first 24 h after injection. Compared to before injection, the pain sensitivity of goats kids was higher 24 h after injection of clove oil and up to at least 6 h after isoeugenol injection, while, in calves, pain sensitivity was highest after 9 h. Future studies should compare the welfare effects of isoeugenol injection with hot-iron disbudding.

**Abstract:**

In this preliminary study, we compared changes in mechanical nociceptive thresholds (MNT) of calves and goat kids injected with clove oil or isoeugenol under the horn bud as a potential, more welfare-friendly alternative to hot-iron disbudding. Twenty male calves and goat kids were randomly allocated to clove oil (n = 10 per species) or isoeugenol (n = 10 per species) injection under the horn buds. MNT was measured via a pressure algometer in calves and kids at several locations around the horn buds at several time points before and up to 24 h after injection. In kids, von Frey filaments were used additionally at the same time points. In calves, linear mixed models revealed an effect on MNT of time point (*p* = 0.010) and side (*p* = 0.007), but not of injection (*p* = 0.298), nor of the interaction ‘injection*time point’, MNT waslowest 9 h post-injection. In goats, there was an effect of injection depending on time point (interaction injection*time point, *p* = 0.03) with MNT being lowest 24 h post-injection for clove oil, while MNT was similar to pre-injection in isoeugenol. In both species, variation in the individual response post-injection was very high. Our results suggest that clove oil and isoeugenol induced hypersensitivity, which was higher for clove oil, in goat kids, but they also suggest a transient anaesthetic effect in some animals and locations.

## 1. Introduction

Disbudding is a widespread husbandry procedure in dairy farming to avoid a potential risk of horn-induced injuries to animals and, especially in cattle, to stockpersons [1,2,3,4]. The most common method used in young calves and goat kids is hot-iron disbudding [5], which leads to burn injury, with associated inflammation, necrosis and haemorrhages, and is a known cause of stress and pain [6,7]. In goat kids, in particular, there is a risk of thermally induced brain damage due to the very thin skull and large horn buds [8]. Thus, the animals would benefit from alternative disbudding methods that offer the potential to reduce the risk of such serious injuries and pain during the procedure.

Clove oil (*Caryophylli aetheroleum*) is an essential oil obtained by distillation of *Syzygium aromaticum* and consists of 36 components, with eugenol being the principal chemical constituent (88%) [9]. Isoeugenol as a pure substance (98% cis- and trans eugenol) is derived from eugenol by a heating process [10]. Clove oil and its main component eugenol, as well as isoeugenol, have anaesthetic and analgesic properties; they can be used locally such as in (human) dental medicine or systemically as is common in fish medicine (for review: [9]; in fish: [11,12,13]; in rodents: [14,15]). Clove oil and (iso)eugenol also exhibit cytotoxic effects: for instance, eugenol was shown to cause apoptosis in cancer cells in vitro and in animal models after different forms of systemic application [16]. Cytotoxic effects of clove oil on human skin cells were caused by eugenol, but not by β-caryophyllene, the second highest concentrated constitute in clove oil [17]. The combination of cytotoxic and anaesthetic effects may make clove oil a preferred alternative to hot-iron disbudding: it may destroy the horn-building cells while inducing only a low level of pain and avoiding the health risks of hot-iron disbudding. In two studies [18,19], the injection of clove oil under the horn bud induced necrosis of the horn bud and was effective in preventing horn growth in five-day-old calves and kids; nonetheless, sample sizes were very low (N = 5) and the authors did not investigate effects on animal welfare. Efficacy was lower in more recent studies, which included larger sample sizes of both calves [20] and goat kids [21]. A small study in calves using clove oil and isoeugenol suggests that efficacy may be dose-dependent [22].

The mechanical nociceptive threshold (MNT) is a measure of the degree of mechanical pressure an animal can tolerate before exhibiting a withdrawal response [23,24,25]. MNT is measured in gram force or Newton applied; the resulting pressure depends on the size of the area on which the force is exerted, and thus depends on the diameter of the probe used for testing MNT. The assessment of MNT can be used to quantify localized effects on sensitivity in the presence of tissue damage (e.g., wound) and to quantify potential subsequent pain, for example in cattle subjected to hot-iron branding [26] or digital dermatitis [24]. The lower the MNT, the higher is the sensitivity with potential implications to the overall pain experienced by the animal. MNT has been used in several studies to assess pain sensitivity around disbudding wounds in calves [23,25,27] or goats [28]. The devices used in these studies were characterized by different maximum cut-off pressure values. Mintline et al. [27] used von Frey Filaments, which, however, did not induce a withdrawal response in many of the calves, even when maximum cut-off values were reached. Heinrich et al. [23] and Stock et al. [25] could identify a decrease in MNT after hot-iron disbudding in calves by use of a handheld pressure algometer (Wagner Force, Wagner Instruments, CT), which allows applying forces of greater magnitude (e.g., in Stock et al. ceiling at 10 kg of force). In goat kids, sensitivity, measured with a similar pressure algometer (Force one FDIX 50, Wagner Instruments), around the horn buds/horn bud wounds was reported to be higher 1 h after application of caustic paste or cryosurgery as compared to hot-iron disbudding, which, however, induced higher sensitivity than sham-disbudding [28]. The question remains open as to whether the injection of clove oil or isoeugenol could be an alternative disbudding method that does not induce such increased pain sensitivity.

To our knowledge, only one study to date has investigated MNT after the injection of clove oil under the horn bud [29]. MNT was measured in calves 24 h before and 48 h after the injection of clove oil, hot-iron disbudding or the application of cautery paste and all these methods were associated with an increase in localized sensitivity. However, the changes in MNT in the first 24 h after injection of clove oil were not investigated. Furthermore, to our knowledge there are no previous studies on the use of isoeugenol as an alternative to clove oil or on the effects of clove oil injection on MNT in goat kids.

The aim of our preliminary study was thus to compare changes in MNT in the first 24 h after the injection of clove oil or isoeugenol in the horn buds of calves and goat kids. We expected both substances to produce an anaesthetic effect within the first one to two hours post-injection followed by an increase in pain sensitivity around the injured sites as the anaesthetic effects vanished. The local anaesthetic effect of clove oil has been demonstrated to last approximately 15 min when applied topically on the cornea of rats [30]. Due to the subcutaneous injection, we expected a longer anaesthetic effect, because the SC injection enables bypassing the barrier of the upper dermis, ensuring that clove oil reaches the deeper dermal structures without the dispersion that characterizes topical applications. As clove oil also contains substances in addition to (iso) eugenol that may also be irritant and induce hypersensitivity [17,31], we hypothesized that there would be higher degree of hypersensitivity and reduced the anaesthetic effects of clove oil compared with isoeugenol.

## 2. Materials and Methods

### 2.1. Animals and Housing

#### 2.1.1. Calves

The experiment took place at the University clinic for ruminants at the Vetmeduni Vienna in November 2016. Twenty male Austrian Simmental calves were bought from a total of 17 farms from Lower Austria and transported together to the Vetmeduni Vienna. They were seven to 14 days old at arrival. The animals were divided into five groups of four animals each and housed in straw-littered pens of 3 × 3 m size, i.e., 2.25 m^2^/animal. Pens were daily littered with fresh straw. The calves were fed milk replacer (Alpmil, Garant-Tiernahrung GmbH, Pöchlarn, Austria) three times a day from buckets. Hay and concentrate were offered ad libitum. Several calves fell ill (diarrhea, respiratory tract infection) three to four days after arrival. They were treated with antimicrobial and/or anti-inflammatory drugs when necessary. As long as feeding behaviour and general condition was not impaired, MNT measurements were taken, and treatment with non-steroidal anti-inflammatory drugs (NSAID, Norocarp, 1.5 mL i.v., the number of animals treated with NSAID equally distributed across treatments and time points) was considered for analysis.

#### 2.1.2. Goats

The experiment was performed in February 2017 in the same barn at the clinic for ruminants at the Vetmeduni Vienna with 20 goat kids. Saanen goat kids were used and all came from one farm; they were 7 to 14 days old on the day of arrival and were split into five groups of four animals each. Each group was kept in straw-bedded pens of 3 × 3 m size, i.e., 2.25 m^2^/animal, which were bedded daily with fresh straw. One corner of the pen was separated by a straw bail and an infrared lamp was installed to create a microclimate zone of higher temperature for resting (ambient temperature 5–10 °C, humidity 52–68%). Goat kids were fed milk replacer (Alpmil Lamm, Garant-Tiernahrung GmbH, Pöchlarn, Austria) four times a day. Depending on their abilities, they were bucket or bottle fed. Hay was offered ad libitum on the ground at one edge of the pen.

### 2.2. Experimental Design and Procedures

#### 2.2.1. Treatments

All procedures involving animals were discussed and approved by the institutional ethics and animal welfare committee in accordance with Good Scientific Practice guidelines and national legislation (BMWFW-68.205/0049-WF/V/3b/16).

The calves and goat kids were randomly assigned to injection with clove oil or isoeugenol (n = 10 per treatment for both calves and goat kids). A total of 1 mL (calves) or 0.2 mL (goat kids) of clove oil (Frey and Lau GmbH, Henstedt-Ulzburg, Germany) or isoeugenol (98% cis/trans Isoeugenol, Sigma-Aldrich Handels GmbH, Vienna, Austria) was injected under the horn bud of calves or goat kids, respectively, medio-rostral from the horn bud, at a 45° angle using a 16G needle (BOVIVET 16G x 1-1/2” 1.6 × 38 mm, Jørgen KRUUSE A/S, Langeskov, Denmark). Calves were injected on the fifth day after their arrival, while goat kids were injected the day after for 10 kids (five per treatment) and two days after arrival for the other 10 kids. In addition to the data on mechanical nociceptive thresholds (MNT), blood samples were collected for residue analysis (data not presented in this report). For this blood sampling, they were catheterised several days before injection (calves) or about two hours before injection (goat kids).

#### 2.2.2. Time Points of MNT Measures

**Figure 1:**
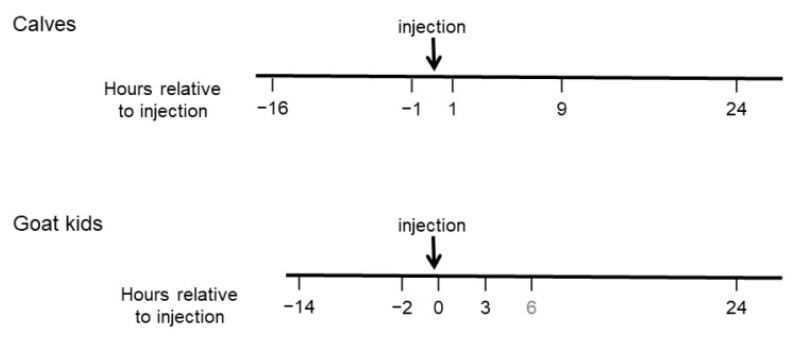
Time points of mechanical nociceptive threshold (MNT) measurements in hours relative to injection of clove oil or isoeugenol in goat kids and calves. Injection was performed around midday one or two days after arrival in goats and around 1 h after feeding in the morning of the fifth day after arrival in calves. The time point 6 h after injection (in kids) is written in grey, as only a few kids were tested due to the late time and thus sleepy animals. Calves underwent a habituation procedure once daily in the three days prior to the first MNT measurement (not depicted).

In calves, MNT were measured approximately 16 and 1 h before injections and 1, 9 and 24 h after injections (Figure 1). Injections were performed in the morning, starting around 1 h after feeding.

Goat kids were injected in two replicates with 10 goats each (five of each treatment), with the first replication starting one day after arrival of the kids at the clinics, and the second replicate starting one day after the first. MNT were measured for both replicates about 14 and 2 h before injection, immediately after injection (0 h), and 3, 6 (in a reduced number of goat kids) and 24 h after injection (Figure 1). Goat kids were injected around midday, so that measuring 9 h after injection (as in calves) was not possible as the goat kids were sleeping. Furthermore, based on our experience with the calves, we aimed at getting data on the course of sensitivity in the first hours after the injection of clove oil and isoeugenol, thus including more time points in the first hours after injection.

#### 2.2.3. Procedure of MNT Measurement

**Figure 2:**
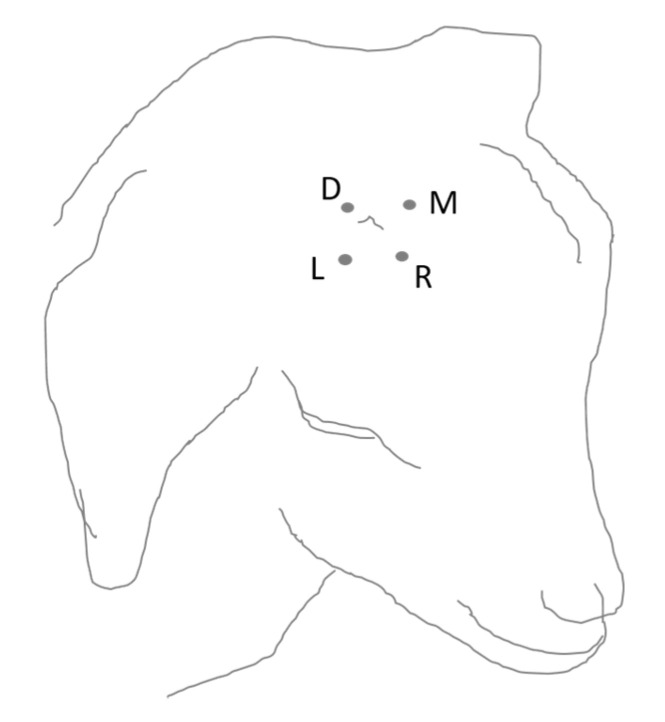
The drawing shows a scheme of the four locations around the horn bud used for MNT measurement (only right horn bud shown on drawing). L, lateral; D, dorsal; M, medial; R, rostral.

All MNT measurements were performed by the same operator (SF), who was blind to the treatment. MNT were measured at four defined locations around the two horn buds, i.e., dorsal, lateral, medial and rostral (Figure 2). To deliver mechanical noxious stimuli, a pressure algometer was used in calves and goat kids and in addition, in goat kids only, von Frey filaments were applied.

Attached to the probe of the pressure algometer (ProdPlus^®^ from Topcat Metrology Ltd., Ely, United Kingdom) was a metal tip of 2 mm in diameter, which was applied at a 90° angle. The pressure was steadily increased at a rate of approximately 2 N/s until the animal exhibited a reaction (withdrawal movement of the head in both species, in goats an ear flick was also considered a withdrawal response) or until the maximum cut-off force of 20 N was reached. If animals reacted at the slightest touch of the pressure algometer with a head movement they were assigned a value of 0.

In goat kids alone, von Frey filaments (Touch Test Sensory Evaluator, Stoelting, Wood Dale, IL, USA) were introduced in addition to the pressure algometer to detect responses to mechanical stimuli of a lower strength. The use of von Frey filaments was decided because of the experiences made with the measurement in calves that were performed three months earlier. The above-described withdrawal responses observed in the calves when the probe was in contact with the application site, but no pressure was exerted, was indicative of mechanical allodynia. We assumed these reactions could be inherently elicited by clove oil/isoeugenol independent of species. Thus, to get more sensitive data, we decided to use von Frey filaments in addition to the pressure algometer in kids. Von Frey filaments allow significantly lower forces to be tested (0.08 mN to 2.94 N, corresponding to the theoretical pressure of 2.53–292 g/mm^2^, compared to the 0.5–20 N corresponding to 50–2040 g/mm^2^ of the pressure algometer).

##### Calves

At each time point, three measurements were recorded per location. To avoid sensitisation, the stimuli were never applied consecutively to the same location, but MNT was assessed at all eight locations once before starting again at the first location. The order of locations was the same in all animals, starting with the right side dorsal, lateral, rostral and medial, then the left side in the same order. The time in between single stimuli at the exact same location was approximately 1.5 min. During measurement, calves were held manually by a person squatting or bending down, the head was held only loosely to permit clear reactions.

The calves were habituated to the procedure during the three days of acclimatization from the arrival to the facility to the date of the first data collection: once a day, the operator applied the pressure algometer on the head around the horn buds (but not at the same locations as for measurements), i.e., touching the skin gently with the tip of the pressure algometer without exerting force.

##### Goats

For the measurements, the goat kids were held in the arms of one experimenter, while another experimenter applied the stimulus and a third person noted down the results. The area around the horn buds was sheared before any recording of measurements could begin (at least 2 h before measurement), and each of the location was marked with a marker.

Von Frey filaments were used first, laterally from each of the horn bud, as described above. The pressure algometer was applied at the dorsal, rostral, and medial locations of the horn bud once per location. A repetition of measurement with the pressure algometer per location was waived in goat kids due to feasibility and to reduce the risk of a learned avoidance.

The measurement with the von Frey filaments was performed according to the up-and-down method [32], always starting with the thinnest filament. The filament was applied at an angle of 90° to the skin, then it was pressed down until it bent (indicating that the appropriate force was applied), pressure was released slowly, and the filament removed. Each application took approximately 2 s. If the goat did not respond to the stimulus, the next thicker filaments, allowing higher force, were applied in succession until a response was observed. When the goat showed a behavioural response (ear twitch, head movement), the filament size was recorded and the next stimulation was carried out with a filament of a two-fold lower force (the second thinner filament). Filaments of increasing force were applied again to induce a second and a third observable response. Thus, three values per location per time point were recorded.

### 2.3. Statistical Analysis

Statistical analysis was performed using SPSS 24 (IBM, New York, NY, USA) by a person blind to the treatment. In calves, the possible side effects were initially analysed by using only the two pre-injection time points by applying a linear mixed model (LMM) with the median of the three measurements per location (e.g., left dorsal) as target variable with fixed effects time point, injection (clove oil, isoeugenol), location (dorsal, lateral, medial, rostral) and side (left, right) and random effect of animal. There was no effect of time point, injection or location, but a significant effect of side (with left < right). Therefore, for the following analyses, the three measurements per location were aggregated by taking the median. LMM were calculated with the same fixed effects and random effect as above, with the addition of the factor NSAID (yes (application Norocarp 2–5 h before measurement), injection one day before (22–27 h before measurement), no (injection at least 48 h earlier)) across all five time points. To fulfil model assumptions (which were checked graphically for all models), the target variable was log-transformed. On the day before injection, five calves (1 isoeugenol, 4 clove oil) had severe diarrhea, and were thus excluded from MNT measurement at time point −16 h. All animals recovered except for one (assigned to the clove oil treatment group), which was excluded from all measurements. To test if intra-individual variation differed between time points, we calculated the standard deviation of the values of the eight locations per time point and animal. This standard deviation (log-transformed to fulfil homoscedasticity) was then used as target variable in a LMM with time point, injection and their interactions and NSAID as fixed effects and animal as random effect. To test specifically for potential differences in anaesthesia, MNT was dichotomized into values from 0–10 and values larger than 10 up to the maximum force. These values were analysed separately via chi-square tests for differences between treatment and differences between locations. The threshold of 10 was selected because all measured values (with one exception) at the two measurement points before injection were clearly below 10. We tested these values on location basis (chi-square test) as well as on an individual basis with dichotomized data (yes: animal did show hyposensitivity at one or more locations, no: no hyposensitivity at any location at the respective time point) by Fisher exact test.

In goats, only the measurements with the von Frey filaments were analysed by an LMM, as only a few measurements were possible where pressure could be applied with the pressure algometer after injection due to head movements at the slightest touch. Thus, we show only descriptive statistics (boxplots) for the pressure algometer data and focussed on the more sensitive vFF-data for inferential statistics. The measured filament sizes were converted into a milli-Newton applied force. The median of the three values per side for the von Frey Filaments were compared in a LMM including injection (clove oil, isoeugenol), time point (1 to 6) and side (right, left) and the interaction time point*injection as fixed effects and animal as random effect. As no side effect was found (F_1,159_ = 1.004, *p* = 0.318), both sides were combined by taking the median of the six values per goat kid for further analysis (fixed effects as above except side). To fulfil model assumptions, the target variable was log-transformed. Due to the late time of injection in goat kids, the measurements 6 h after injection were performed only in three (clove oil) or four (isoeugenol) animals, but then stopped due to animals becoming drowsy. Some animals were sick at one or two of the time points (diarrhea, respiratory tract infection) and were not tested, reducing sample size (see Figure 4 for exact sample size). All graphs are based on original data. All values given in the text are based on estimated means and 95% confidence intervals (CI) derived from the LMM models. Because log-transformation of the target variable was necessary, the values are back-transformed.

## 3. Results

### 3.1. Calves

**Figure 3:**
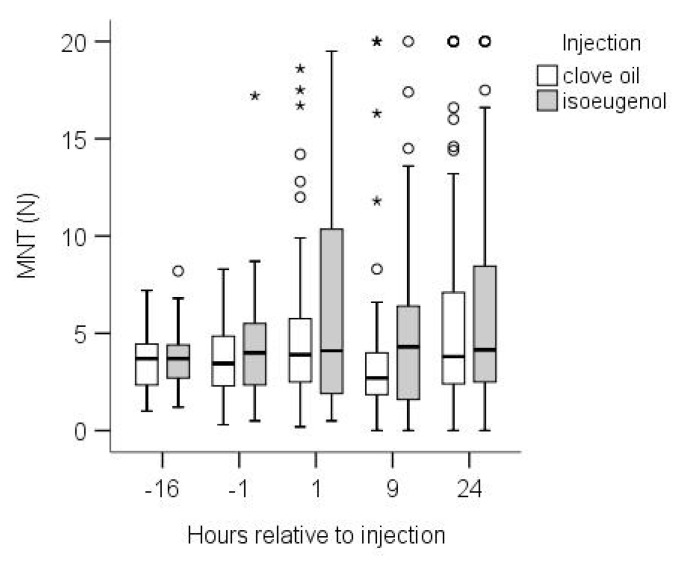
Mechanical nociceptive threshold of the 20 calves on the five time points (TP) the day before, i.e., about 16 h before (−16), injection of clove oil (white boxes) or isoeugenol (grey boxes), on injection day 1 h before (−1), about 1 (1) and 9 h (9) after injection, and 24 h after injection (24). The value 20 N represents the maximum; the value 0 was assigned if no measurement was possible due to avoidance reactions at slightest contact. Graph is based on original data, median value per location, i.e., 8 points per animal (Isoeugenol: N = 72 for TP-16, N = 80 for all other TPs; clove oil: N = 48 for TP-16, N = 72 for all other TPs). The Box–Whisker-Plot shows median (bar in box), 25 and 75% quartile (bottom and top end of the box) and minimum and maximum (whiskers) except for outliers (circles, distance to box 1.5–3 times interquartile range IR) and extreme values (asterisks, distance to box >3 times interquartile range).

The LMM revealed an effect of time point (F_4,693_ = 3.350, *p* = 0.010, Figure 3) and side (F_1,680_ = 7.307, *p* = 0.007), but not of injection (F_1,16_ = 1.153, *p* = 0.298, Figure 3), location (F_3,679_ = 1.266, *p* = 0.285) or NSAID (F_2,505_ = 1.244, *p* = 0.298) on MNT, nor of interaction between injection and time point (F_4,686_ = 0,697, *p* = 0.594). Values were slightly higher on the right (estimated means, 95%CI: 3.8; 3.23–4.48 N) than on the left side (13.27, 2.78–3.85 N). Comparing time points, MNT was lowest 9 h after injection (2.93, 2.32–3.71 N), and highest 24 h (4.07, 3.34–4.96 N) after injection, with a significant difference only between these two TPs (*p* = 0.021). The other TPs did not differ significantly (−16 h, 3.44, 2.80–4.22 N; −1 h, 3.37, 2.78–4.09 N; 1 h, 3.93, 2.23–4.78 N). Individual variability appeared to increase following injection. (Figure 3). Accordingly, LMM on the standard deviation revealed an effect of time point (F_4,661_ = 12.189, *p* < 0.001). Variability (standard deviation) was higher 1 h (3.42, 2.54–4.61) and 24 h (3.31, 2.49–4.40) post-injection as compared to the two time points before injection (−16h: 1.15, 0.83–1.58; −1 h: 1.48, 1.11–1.98; all four *p* ≤ 0.001); 9 h post-injection, variability was higher (2.19, 1.51–3.18) compared to 16 h before (*p* = 0.028) but not compared to 1 h before (*p* > 0.05) injection. Variability did not differ within pre-injection time points or within post-injection time points.

A MNT > 10 N (reflecting very low sensitivity) was measured more often in isoeugenol-injected calves (48 out of 344, i.e., 12.2%) than in clove-oil-injected calves (24 out of 320, i.e., 7%; Chi^2^ = 5.762, *p* = 0.016). This was primarily due to the higher occurrence of MNT > 10 N at TP 1 h after injection (isoeugenol: 20 out of 80, i.e., 25%, clove oil: 6 out of 72, 8.3%; Chi^2^ = 7.424, *p* = 0.006), while no difference was seen 9 h (isoeugenol: 11/80, 13.8%; clove oil: 5/72, 6.9%) and 24 h later (isoeugenol: 16/80, 20%; clove oil: 13/72, 18.1%). There was no difference in the number of animals that did show hyposensitivity at one or more locations at any time point (TP-16, −1, 1, 9, 24: isoeugenol 0, 1, 7, 5, 5; clove oil 0, 0, 5, 2, 5), i.e., locations with hyposensitivity were not clumped in individual animals but scattered over the animals at TP 1h.

### 3.2. Goats

**Figure 4:**
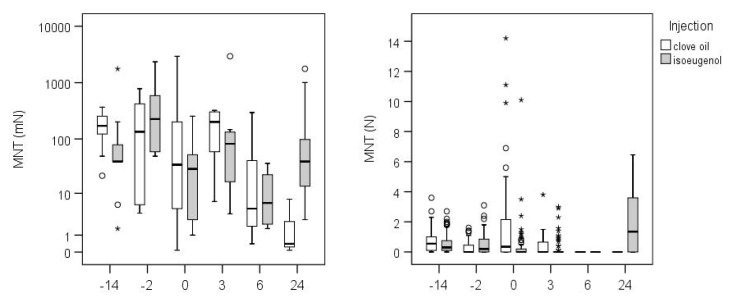
Mechanical nociceptive threshold, measured by von Frey filaments (vFF, **left**) or pressure algometer (PA, **right**), of the 20 goat kids on the six time points (TP) the day before, i.e., about 14 h before (−14), injection of clove oil (white boxes) or isoeugenol (grey boxes), on injection day 2 h before (−2), immediately after injection (0) and 3 h (3) and 6 h (6) after injection, and 24 h after injection (24). Graphs are based on original data, please note the different scales: The values of vFF represent target force in mN on a logarithmic scale (ranging from a minimum of 0.08 mN to a maximum of 2.94 N); values of PA are shown in applied force in N. For vFF the median of all six measures per time point (clove oil: N = 10, 10, 9, 9, 3, 3 for TP-14, −2, 0, 3, 6, 24; isoeugenol: N = 9, 10, 10, 10, 4, 9) is shown, for PA the individual measurement points (i.e., 8 values per animal) are included, N see vFF except for TP 6 and 24, clove oil: N = 2, 2; isoeugenol, N = 4, 6). The Box–Whisker-Plots show median (bar in box), 25 and 75% quartile (bottom and top end of the box) and minimum and maximum (whiskers), except for outliers (circles) and extreme values (asterisks).

There was an effect of injection depending on time point (interaction time point*injection: F_5,68_ = 2.585, *p* = 0.033) as well as a main effect of time point (F_5,68_ = 5.690, *p* < 0.001), but no main effect of injection (F_1,20_ = 1.226, *p* = 0.281). On the day of injection, the course of MNT was similar for clove oil and isoeugenol while, at the day after differences occurred, immediately post-injection, MNT was lower than before in both treatments (Table 1, Figure 4). It increased again three hours later to reach the starting level recorded the day before injection, but decreased six hours after the injection for both treatment to a level about 10-fold lower than pre-injection levels (Table 1). Twenty-four hours after the injection of clove oil, a 100-fold lower force was necessary to elicit reactions compared to before, i.e., kids experienced a 100-fold higher sensitivity, whereas MNT was only slightly lower 24 h after isoeugenol injection (Table 1, Figure 4). In the clove oil treatment, a large variation is striking directly after injection (TP0) ranging from the lowest possible force (0.08 mN) to the highest possible force (2940 mN, Figure 4, Table 1). This high variation is also visible in the data of the pressure algometer, shown in Figure 4, where animals show the highest MNT values at time point 0, ranging up to 14 N. The difference between clove oil and isoeugenol 24 h after injection is also reflected in the pressure algometer data (Figure 4).

## 4. Discussion

In this study, we compare changes in mechanical nociceptive threshold in the first 24 h after the injection of clove oil or isoeugenol for disbudding in calves and goat kids. In both species, the variability of MNT increased markedly after injection, not only with large inter-individual differences but also within individuals and across locations. The results suggest a local anaesthetic effect (MNT increased to the maximum force possible without inducing a response) as well as hyperalgesia/allodynia (lowest measurable MNT or even no full measurement possible with the pressure algometer due to response to touch with no applied pressure) after injections of clove oil or isoeugenol. In addition, we detected differences in post-injection MNT development over time that were more prevalent in goats.

### 4.1. Calves

We found an effect of time and side on the MNT for both isoeugenol and clove oil injection, but no difference between the two treatments. Compared to the measurements before the injection, the data collection was more difficult after injection, since the animals partly withdrew the head at the minimal contact with the measuring device, i.e., before applying any pressure. Consequently, several attempts were necessary to be able to obtain one measurement. Failure to take a complete measurement corresponded to a value of 0. On the other hand, after the injection calves were very insensitive at some locations, i.e., they did not react with head movement, even when the maximum cut-off force was reached. Thus, a high level of variability of MNT, ranging from 0 to the maximum of 20 N, was found for all time points after injection, and variability was larger after injection than before, especially at time points 1 and 24 h post-injections. The insensitivity of calves at some locations, i.e., the complete absence of a withdrawal response even following the application of the maximum possible mechanical pressure, seems to confirm previous observations on the anaesthetic effect of (iso)eugenol (e.g., [9,11,13,14]). The anaesthetic effect/insensitivity was most pronounced one hour after injection, although it was also observed 24 h later across several locations and animals. To our knowledge, no previous study has investigated the duration of the local anaesthetic effect of clove oil or isoeugenol/eugenol. Ghelardini et al. [33] tested the local anaesthetic effect of ß-caryophyllene in rabbits: local anaesthetic activity started 5 min and disappeared within 15 min of application into the conjunctival sac. In humans, clove gel has been reported to exert a topical anaesthetic effect to a needle prick, comparable to benzocaine gel, five minutes after application to the buccal fold [34]; however, the duration of the effect was not tested. Regarding systemic effects, Guenette et al. [14] observed anaesthetic activities in rats with a mean recovery in reflex time of 167 s after i.v. injection of eugenol at a dose of 60 mg/kg, and of about 15 s after the administration of 5 mg/kg.

The mechanisms behind the insensitivity observed in this study 24 h after injection warrants further investigation to elucidate whether this is the result of either a long-lasting direct anaesthetic effect following subcutaneous injection, or of tissue destruction, including nerves, due to the cytotoxic effects of clove oil and isoeugenol. To support the latter explanation, we observed calves developing necrotic areas visible in dark colouration some days after the injection. A systemic effect of eugenol or isoeugenol after resorption from the injected area is very unlikely, because the anaesthetic effect was unevenly distributed between locations within one animal (see also below).

Although there were no significant differences between treatments in general sensitivity, isoeugenol appeared to exert a stronger anaesthetic effect than clove oil. We recorded a greater incidence of ‘no responses’, i.e., no reaction to pressure higher than 10 N, after the injection of isoeugenol than clove oil, especially shortly after injection. To our knowledge, this is the first attempt at comparing the anaesthetic effectiveness of clove oil and isoeugenol.

Despite detecting insensitivity or reduced levels of sensitivity post-injection in limited numbers of animals and locations, we also recorded a simultaneous increase in sensitivity at other locations at the same time points, even within the same animals. This suggests the development of hyperalgesia or allodynia in these areas with potential implications to pain experienced by the animals. The remarkable intra-individual differences between locations, even within the same horn bud side, ranging in some animals from a value close to the minimum up to the maximum force, may suggest that the injected substances were not absorbed or did not disperse consistently in the whole area around the horn bud, possibly reducing the anaesthetic acting dosage at some locations. Further investigation is required to elucidate whether the cytotoxic effect needed to destroy the horn building cells is also dependent on the uniform distribution of the substance at all locations. In recent studies involving both calves and goat kids, clove oil injections under the horn bud were not sufficiently effective in preventing horn growth. They instead led to abnormal horn growth (scurs), suggesting insufficient destruction of the germinal tissue [20,21], which might be a result of the inconsistent distribution of the drug.

Approximately half of the animals in this study responded to the mechanical challenge with large intra-individual differences. Animals adopt different coping strategies when dealing with challenges [35,36,37]), which include different degrees of sensitivity to painful events [38]. In addition, there may be differences in individual sensitivity to isoeugenol and clove oil or to some of their components.

Post hoc comparisons indicated that MNT following injection did not differ significantly from the baseline measurements collected before inducing the injury. Any overall effect of the injection on changes in mechanical sensitivity over time may have been concealed by the high individual variability post-injection, which resulted in animals displaying either increased or decreased response thresholds, regardless of the treatment. Sutherland et al. [29] measured MNT in calves 24 h before (baseline) and 48 h after the injection of clove oil or hot-iron disbudding. Compared to baseline, pain sensitivity was higher 48 h post-procedure in clove oil- and hot-iron treated calves, but in clove-oil-treated calves this was significant only in one of the eight locations measured around the horn buds, while, in the hot-iron treatment, this was the case for six of the eight locations. Their observations appear to confirm a similar degree of variability after clove oil injection as that observed in this study. Nonetheless, Sutherland et al. do not report absolute values, therefore making a direct comparison unfeasible. This may also suggest that the anaesthetic effect vanishes in approximately 24 to 48 h, leading to a generally higher sensitivity than before.

We did not find an effect of carprofen on MNT. While NSAID may reduce hyperalgesia, the effect depends on the specific drug used, on the way of application (locally or systemically) and the type of noxious stimulus [39]. Our results are in line with studies showing a minimal to no effect of carprofen on MNT in calves after hot iron disbudding or on MNT in healthy cats [40,41]. Although the number of animals treated with carprofen was equally distributed across treatments and the response patterns strongly suggest the lack of an influence of carprofen on mechanical sensitivity after injection of clove oil or isoeugenol, we cannot exclude the presence of an unwanted effect.

### 4.2. Goats

The effects of the injection of clove oil and isoeugenol differed depending on time point. While both increased sensitivity in the first day of injections, with the exception of a brief reduction in sensitivity demonstrating an anaesthetic effect 3 h post-injection, only clove oil caused an even enhanced increase in sensitivity up to 24 h after injection. Thus, clove oil seems to induce hyperalgesia and allodynia in goat kids that is more sustained compared to isoeugenol. However, data of the clove oil treatment 24 h after injection need to be interpreted cautiously due to the very small sample size. Nevertheless, it is conceivable that clove oil, which comprises 36 different substances, may have more irritating properties compared to isoeugenol as a pure substance. While MNT was lower immediately after injection of both substances, it returned to baseline levels three hours after injection. This may be due to an uneven dispersion or absorption of the substances through tissue, which may have resulted in a slower onset of local anaesthesia, exceeding the 10–15 min measurement period. As previously discussed, there is only evidence of an immediate (approx. 5 min) anaesthetic effect of clove-oil delivered topically in rats and humans. Therefore, the unprecedented subcutaneous application described in this study requires a cautious interpretation of the results in the context of the existing literature.

In order to avoid exposing the animals to excessive handling and restraint, we reduced testing time by applying the von Frey filaments at one location per side only. Subsequently we combined both sides into one value for further analysis. With the exception of the measurements obtained from clove oil treated animals directly after injection, we observed a reduced level of variability in the data compared to calves. This difference may be explained by the low number of data points obtained from the goats, which is in contrast with the eight locations per time point analysed separately in the model applied to calves’ data.

Due to some calves developing signs of allodynia at least at some locations, we deliberately chose to use von Frey filaments in goat kids, which exerts forces of considerably lower magnitude. Indeed, in a high proportion of animals it was not possible to measure MNT with the pressure algometer following the injection. Baseline MNT values appeared to be generally lower in goat kids than in calves, suggesting a greater degree of mechanical sensitivity in the former (data not shown).

### 4.3. General Discussion–Limitations of the Study

The increased sensitivity at some locations and time points in both calves and goats as well as after both clove oil and isoeugenol injection may indicate a potential irritation by the injected substances if the anaesthetic dosage is not reached. However, we cannot exclude that the injection itself (being composed of the needle prick plus injection of a liquid under the horn bud) may have affected sensitivity as this experiment did not include a control treatment exposed to the injection of a non-harmful solution (e.g., saline). A comparison of sensitivity with control animals was not in the scope of this preliminary project. To draw any conclusion about the effects on animal welfare, further studies are required that incorporate a true control group as well as the additional behavioural and physiological indicators of pain induced by disbudding. While the injection of clove oil or isoeugenol is advantageous compared to hot-iron disbudding in terms of imposing no risk for thermal damage to the skull and brain, potential damage by the cytotoxic effects of the substances needs to be investigated before drawing firm conclusions.

The occurrence of illness was quite high. For the goats in particular, this resulted in a substantial reduction in sample size at some time points. In calves, several animals were treated with NSAID. Grouping animals from different farms (calves in particular), transport and transfer to a novel environment are well-known risk factors for disease [42], which could not be avoided.

In principle, the handling of animals during the delivery of mechanical pressure may induce stress, potentially affecting the outcome [43,44]. However, this depends on the existing animal–human relationship [45] and the procedure implied. Calves and goat kids used in this study were bucket fed from birth, which is a practice known to benefit handling, as demonstrated by the ease with which they approach humans [46,47]. All animals were calm during handling and thus could easily be held during MNT measurement. Calves were habituated additionally to the experimenter and the MNT measurement procedure the days between arrival and start of the experiment. During habituation, gentle tactile contact was deliberately imposed in order to further improve their relationship with humans [48]. Furthermore, we tested them in their home pen close to their peers, avoiding stress due to novel environment or isolation. In sum, we trust that these actions have been effective in minimizing any stress-induced alterations in the MNT recordings.

## 5. Conclusions

The results of the measurement of the mechanical nociceptive threshold in calves and goat kids suggest that injections of clove oil and isoeugenol for the purpose of disbudding may lead to the development of mechanical hypersensitivity (hyperalgesia or allodynia). In addition, the two substances appear to induce anaesthesia or hypoalgesia, although these changes are location and time-point-dependent. In comparison with clove oil, isoeugenol offered the advantage of inducing a more consistent anaesthetic effect and, at least in goats, less sustained hypersensitivity, which resolves 24 h after injection. Further research is necessary to compare the effects of isoeugenol and clove oil injection with those of NaCl injections and hot-iron disbudding.

## Figures and Tables

**Table 1:** Results of the linear mixed model for MNT measured with von Frey filaments in goat kids illustrating the interactions time point*injection (*p* = 0.033). Values are back-transformed estimated means (EstM) and lower and upper limit of the 95% confidence interval (CI) of the force applied (in milliNewton) with vFF on the six time points (TP) about 14 h (−14 h) and 2 h (−2 h) before injection of clove oil or isoeugenol, immediately after injection (0 h) and 3, 6 and 24 h after injection.

Time Point	Clove Oil			Isoeugenol		
	N	EstM (mN)	95% CI (mN)	N	EstM (mN)	95% CI (mN)
−14 h	10	140.63	38.01–520.34	9	50.89	12.88–201.14
−2 h	10	72.73	19.65–269.10	10	222.96	60.26–824.98
0 h	9	19.75	5.00–78.05	10	14.92	4.03–55.20
3 h	9	105.51	26.68–417.30	10	59.76	16.15–221.11
6 h	3	6.79	0.66–70.02	4	10.73	1.42–81.08
24 h	3	0.80	0.08–8.21	9	47.58	12.04–188.03
